# Governing the mixed health workforce: learning from Asian experiences

**DOI:** 10.1136/bmjgh-2016-000267

**Published:** 2017-04-07

**Authors:** Kabir Sheikh, Lakshmi K Josyula, Xiulan Zhang, Maryam Bigdeli, Syed Masud Ahmed

**Affiliations:** 1 Public Health Foundation of India, New Delhi, India; 2 Previous affiliation: Indian Institute of Public Health, Hyderabad, Public Health Foundation of India; Present affiliation: The George Institute for Global Health, Hyderabad, India; 3 China Institute of Health, School of Social Development and Public Policy, Beijing Normal University, Beijing, China; 4 Past: Alliance for Health Policy and Systems Research, WHO; Present: Department of Health Systems Governance, Policy and Aid Effectiveness, World Health Organization (WHO), Geneva, Switzerland; 5 Centre of Excellence for UHC, James P Grant School of Public Health, BRAC University, Dhaka, Bangladesh

## Abstract

Examination of the composition of the health workforce in many low and middle-income countries (LMICs) reveals deep-seated heterogeneity that manifests in multiple ways: varying levels of official legitimacy and informality of practice; wide gradation in type of employment and behaviour (public to private) and diverse, sometimes overlapping, systems of knowledge and variably specialised cadres of providers. Coordinating this mixed workforce necessitates an approach to governance that is responsive to the opportunities and challenges presented by this diversity. This article discusses some of these opportunities and challenges for LMICs in general, and illustrates them through three case studies from different Asian country settings.

**Key questions**

**What is already known about this topic?**

- Health workforce heterogeneity in many low and middle-income countries (LMICs) manifests as variations in level of state recognition, form of employment, system of knowledge and specialisation.

**What are the new findings?**

- Lay workers, informal providers and non-allopathic practitioners in many LMICs are often closest to communities most in need of services. Yet, paradoxically, global and national policies seldom focus adequately on these sections of the workforce.

**Recommendations for policy**

- Goals of improved population health necessitate policies that recognise the *de facto* division of labour among different segments of the workforce, epistemic divergences and unique administrative needs of different cadres.
- Experiences of governance of the mixed health workforce in Asia hold key lessons for comparable pluralistic health systems globally.

## Introduction

The persisting and emerging public health challenges facing the developing world constantly underscore the significance of people-centredness in health systems.^1^ While people-centredness in health systems has largely been synonymous with the needs and preferences of users and populations, it also signifies the people who make up the system—particularly healthcare personnel as the key people on the frontlines of the health system.^2^ The health workforce is embedded in and shaped by societies and markets, and there is a profound need for more informed and contextualised approaches for its governance.

Probably the most striking feature of the composition of the health workforce in many low and middle-income countries (LMICs) is the deep-seated heterogeneity that manifests in multiple ways: varying levels of official legitimacy and informality of practice; wide variation in type of ownership and behaviour (public to private); diverse, sometimes overlapping, systems of knowledge and different cadres of providers, specialised to different extents. Different sections of the health workforce are diverse in their characteristics and needs, and are also connected, in complex ways, with each other and with communities through social and economic networks. Governing this mixed workforce towards a common purpose is central to achieving population health goals. This requires policies that are tailored to the strengths and weaknesses of different sections of the workforce, and responsive to the particular opportunities and challenges presented by its heterogeneity. However, commonly used frameworks describing the health workforce^3–5^ seldom adequately encompass the true complexity of the sector, and the full range of health workers potentially involved in advancing the achievement of population health goals. This knowledge gap contributes to deficiencies in the way that the health workforce is governed.

This paper characterises workforce diversity and complexity in a manner that we hope can help improve its governance. It was developed following participation by the authors on a subplenary panel on health workforce heterogeneity in Asia, at the 2nd Health Systems in Asia conference (December 2013), where panellists presented case studies highlighting the roles of, and the challenges faced by, various sections of the health workforce in different Asian countries. While the literature and cases presented in this paper are from Asian countries, we recognise that a number of LMICs across South and Central America, Africa and Eastern Europe have a similarly heterogeneous health workforce, with private healthcare in particular growing in many LMICs globally. As such, we believe that it has resonance beyond Asia, and represents learning for comparable contexts globally.

We start by presenting a framework for understanding health workforce heterogeneity, developed based on the available Asian literature. Next, we present case examples of three significant sections of the health workforce to illustrate the unique governance challenges that confront them. The final section summarises some of the crucial opportunities and challenges posed by workforce heterogeneity, and outlines recommendations for policy.

## Making sense of health workforce heterogeneity

The composition of the health workforce in the mixed public–private health systems of many Asian countries is deeply heterogeneous.^6–10^ Health workers provide care through a range of public and private facilities, including hospitals, clinics, pharmacies, mobile units and village outposts. In many countries, a large proportion of the workforce lies outside the purview of the formal mainstream health system, and is variously described as the informal, unregulated or unrecognised sector.^11^ ^12^ While many providers of allopathic care, and traditional, complementary and alternative medicine (TCAM) are trained and qualified, there is also a large workforce of unqualified and variably trained providers in both sectors.

For example, according to an estimate in 2007, traditional healers constituted around 44% of the total active healthcare providers in Bangladesh, followed by traditional birth attendants (23%), village doctors (8%), drug shop attendants (8%) and community health workers (6%).^13^ A study from West Bengal, India, found that 54% of the population with any illness was treated by unrecognised ‘rural medical practitioners’.^14^ In a study exploring the role of private providers in China, prior to China's implementation of its healthcare reform agenda, 60% of the rural population was found to seek care from village health posts, staffed by former ‘barefoot doctors’.^15^ In Vietnam, private sector providers (including traditional healers and various allopathic providers) were found to provide basic health services in 60% of rural care seeking instances.^16^ In Cambodia, attendants at drug shops, traditional healers and traditional birth attendants were reported to serve around 20% of the rural population.^17^ According to a World Bank estimate, in Indonesia, 45% of the population relied on self-treatment by buying drugs from shops during their last illness.^18^

### Multiple dimensions

**Figure 1:**
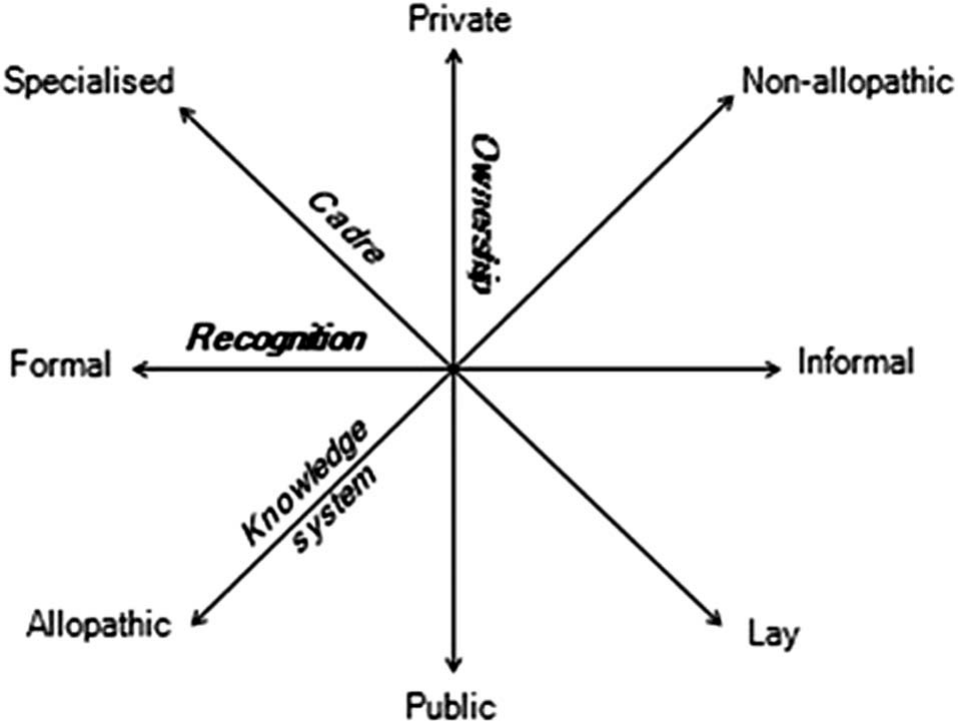
A framework to understand health workforce heterogeneity.

It is evident that health workforce diversity has multiple overlapping dimensions. Figure 1 below describes four key dimensions of health workforce diversity on a conceptual map: ownership (representing a continuum from public to private), recognition (from formal to informal), knowledge system (from Western or allopathic to non-allopathic) and cadre (from specialised to lay). We elaborate and substantiate the understanding of each of these different dimensions in this section.

### Dimension 1: ownership: public to private

Although the focus of health system decision-makers and the public health community is often on public sector programmes, the private sector plays a large, and vital, role in healthcare provision in many Asian countries. For instance, the Cambodian Demographic and Health Survey 2010 showed that 57% of the population sought their first treatment in the private sector;^19^ and Cambodia reported a flow of 68% of out-of-pocket expenditure by patients to the private sector.^20^ The public sector is often preferred for severe illnesses and acute conditions, while patients with chronic conditions tend to prefer the private sector.^21^ The private healthcare sector also includes non-profit providers, who deliver services through varied free or discounted facilities.

Type of ownership of a healthcare facility (public or private) is often associated with overt distinctions in provider behaviour, and there have been numerous studies on and debates over which sector provides better quality healthcare.^22^ However, when we unpack the different ways in which (and degrees to which) healthcare providers are associated with government, this public–private mix may more usefully be seen as a continuum than a dichotomy. A spectrum of such mixes exists, from comprehensive financial, infrastructural and technical provision by the government, to combinations of public and private provision, where, for instance, publicly provided health insurance can be used to receive healthcare at private facilities, to totally privately paid healthcare.^23^ The public–private distinction is also actively blurred in a range of settings, such as informal payments for services and referral from public to private facilities, formal public–private partnerships and service contracts, private practitioners sometimes being associated part time with government hospitals and the widespread phenomenon of dual practice, for example, a government doctor running a private clinic after work hours.^23^

### Dimension 2: recognition: formal to informal

The formal health workforce in many countries is matched by a significant presence of providers who are unrecognised, or only partially recognised, by the state. The majority of the population in most Asian countries lacks access to the formal healthcare system, especially in rural areas, and seeks care from providers in the informal sector, who are frequently more accessible, affordable, responsive and can be more culturally aligned to their lifestyles.^9^ ^24–26^

The healthcare sector in many contexts includes a varied mix of informal providers, such as drug sellers, and untrained—or partially trained—practitioners. Statistical data on the informal health sector in developing countries are particularly scarce; however, there are indications that the services of informal providers are widely used, resulting in well-established, if sometimes illegal, markets of goods and services.^9^ ^27–29^ For instance, in Bangladesh, it is reported that traditional and informal providers operate alongside private for-profit and non-governmental organisation (NGO) providers, with wide variations in population reach and quality of services.^30^ In India, informal providers, who are over half the population of healthcare providers, provide ∼70% of primary healthcare in rural areas.^31^ A provider mapping study in the state of Madhya Pradesh, in India, showed that 30% of all private providers were in the informal sector.^32^

Informal healthcare providers vary widely by duration and nature of training received—many are untrained or informally apprenticed. Some receive instruction from unaccredited institutions that do not follow standard curricula. NGOs have also been known to be involved in training informal providers.^24^ Informal providers are often socially entrenched in the communities in which they live and work, and are often the first point of care, particularly for the poor, owing to their proximity, low and flexible payment structures and cultural affinities. Studies have also revealed the presence of networks between informal and formal providers ranging from apprenticeship, and referral, to paid assistance by the informal provider in busy practices of formal providers.^29^ Increasingly, there are calls to include informal providers in formal health programmes and care networks aimed at enhancing population health access.^33^

### Dimension 3: knowledge system: allopathic to non-allopathic

A poorly understood aspect of mixed Asian health systems is the prevalence of TCAM systems, and their interface with the (typically mainstream) system of western or allopathic medicine.^34^ These include various national traditional medicine systems, for example, traditional Chinese medicine, ayurveda, acupuncture, yoga, meditation, chiropractic and herbalism. Several indigenous medical systems have achieved legal recognition and state support in some Asian countries, for example, Laos, India and Vietnam.^35–37^ Yet, in general, very modest progress has been made in effectively using indigenous health practitioners in healthcare delivery systems at scale, and these practitioners frequently function outside the ambit of government-recognised policies and structures.^9^ ^38^ ^39^

TCAM systems are not neatly separable from allopathic practices. The origins and epistemologies of TCAM variously bear similarities or marked differences with allopathic medicine.^40^ TCAM systems often overlap and converge with the practices of allopathic practitioners through the incorporation of TCAM practices, including prescription of meditation, diets and physical activity regimen, nutritional supplements and tonics by allopathic practitioners.^41^ ^42^ On the other hand, increasing adoption of biomedical terminology and technology is observed in the practice of several TCAM systems to describe clinical cases and arrive at diagnoses. ‘Cross-practice’, or the prescription of medications and therapeutic techniques of a particular system of medicine by a practitioner not trained in that particular system, is a common and often contested practice,^43–46^ and represents another informal type of convergence.

### Dimension 4: cadre: specialised to lay

Whereas advanced tasks and leadership roles in healthcare are typically assigned to professional cadres such as physicians, nurses and pharmacists, healthcare tasks and the division of labour in health systems are continually evolving in response to health worker availability, healthcare needs, technological changes and political factors. Changing the cadre of health workers involved in specific health tasks has been a major focus of global public health policy in recent years. In many cases, such ‘task shifting’ arose in response to shortages of doctors and nurses in rural areas.^47^ Governments began experimenting with training and placing lower level healthcare providers such as medical and nurse assistants in rural clinics. Public primary healthcare in many Asian LMICs is increasingly being provided by community health workers, and upgraded frontline healthcare workers, as in the case of community health workers performing disease-preventive, health-promoting activities as well as basic curative care for common illnesses in Bangladesh.^48^ Further, with rapidly ageing populations in many LMICs, particularly in Asia, the importance of home-based care providers, while inadequately researched, cannot be understated.^49^

Accompanying their greater responsibilities in healthcare and public health, frontline and community health workers are increasingly using opportunities for training and leadership within health programmes, and have also been observed to self-organise to promote their collective interests.^50^ The emerging importance of community health workers, who are often located closest to, and drawn directly from, communities in need, raises questions about disproportionate investments by governments in training and supporting more professionalised or specialised cadres of health workers.

### Policy attention anomaly

The multidimensional representation of health workforce heterogeneity in figure 1 highlights several realities about the identity and behaviour of healthcare providers. In the first place, it is notable that healthcare providers are more usefully described through more than one descriptor, for example, non-allopathic doctors can have state recognition, and/or be highly specialised, or not. Second, diversity along each dimension may be better understood as continua rather than dichotomies. Different health workers are situated at different points along these ‘axes’, for instance, allopathic doctors may integrate elements of traditional medicine into their worldview and practice, just as non-allopathic healers draw from biomedical knowledge systems. Further, a health worker may transition into a different type over the course of time, such as when a physician working in a public clinic in the morning ‘moonlights’ in private practice in the evenings.

**Figure 2:**
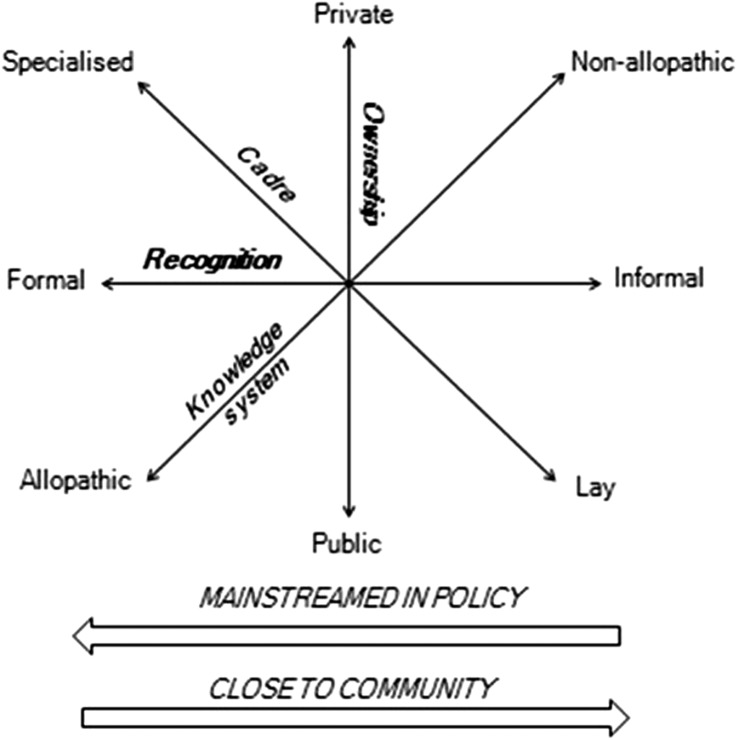
The mixed workforce policy attention anomaly.

A further critically important reality is what could be termed a policy attention anomaly when it comes to the mixed health workforce (figure 2). Sections of the health workforce to the right of the figure, often amounting to a large proportion of the total national workforce, generally lie outside the policy mainstream. Formal global and national policies are seldom drawn up with these sections of the workforce in mind. Yet, paradoxically, these sections of providers are typically (physically, socially and culturally) closest to, and most embedded in, the communities most in need of services, and hence often in an advantageous position to have an impact on population health. Healthcare providers who are easiest to access, and offer flexible payment structures are, frequently, informal providers of a variety of health services, lacking training and government support, and conducting unregulated practices, raising concerns of safety and quality of care.

In the following section, we use case examples to discuss how certain sections of the health workforce in different Asian settings can be located in the framework, and some peculiar governance challenges that confront them.

## Three Asian case studies

Management of a diverse workforce raises complex challenges, and also includes specific opportunities. Beyond creating a platform for physical access to services and financial protection in many Asian countries, national movements and initiatives such as Universal Health Coverage potentially contain opportunities to muster the strengths of these divergent groups of providers towards a common cause. The following case studies, of particular categories of healthcare providers in three different Asian countries, illustrate some of these challenges and opportunities.

### Identity dilemma of village doctors in China

The rural health system in China has a three-tiered structure: county hospitals, township health centres and village clinics.^51^ Village doctors, formerly called ‘barefoot doctors’, have been serving the village clinic level of the three-tiered health system of China for about four decades.^52^ ^53^

Until the 1980s, the barefoot doctors conducted medical services and farming, and their income was derived from local collective economics.^53^ From the 1980s onwards, China's market-oriented social–economic reforms shifted village doctors along the ownership dimension, first from public to private, and then to a public–private mix.^53^ Reform in the 1980s shifted them into the private sector, with their salary no longer being set by the government, but being based on market competition. This reform led these health workers to leave behind their ‘half farmer, half doctor’ identity and mixed income source to become full-time healthcare providers.^54–56^ In 2009, an additional reform exploited the availability of village doctors in villages, and shifted them back to the public sector, dictating that they provide public health services, and sell essential drugs without a markup.^52^ ^57–59^

However, these shifts in the identity of the village doctors were not always supported by corresponding adjustments in regulatory and governance arrangements, leading to challenges for the village doctors. China's social welfare system classifies citizens into four groups: public employees, enterprise employees, urban residents and rural residents.^60^ Despite working full-time as healthcare professionals, and providing a range of public services, village doctors are still identified as farmers, and are thus only entitled to benefits for rural residents. Village doctors asked to be considered public employees, and thereby eligible for the public bonus. The government, however, argued that village doctors remain outside public regulatory authority, and may not always demonstrate high quality in the performance of their tasks. This classification debate continues to keep village doctors at a disadvantage.

The educational and developmental needs of village doctors have also presented vexed challenges. The first challenge was the issue of accreditation. The Ministry of Health of China requires all doctors, except village doctors, to pass the National Licensed Doctor Examination before they are allowed to practise. Village doctors can practise medicine right after obtaining the Village Doctor Certification, issued by county health bureaus. In 2011, only 14% of village doctors passed the National Licensed Doctor Examination.^57^ ^61^ While mandating that village doctors pass the National Licensed Doctor Examination before practising would shift the cadre up the axis of specialisation and professionalisation, and thereby putatively contribute to improving health service quality and safety in rural areas, it would also curtail rural users' access to those providers who remain unlicensed.^62^ The choice and establishment of appropriate education standards to balance the goals of service quality and workforce adequacy is an urgent governance task for policymakers.

The second governance challenge is related to quality improvement, specifically whether existing village doctors should be retrained or the workforce revamped with the introduction of younger and better-educated medical graduates. Almost 60% of practising village doctors have completed only junior college, which is equivalent to a total of 14–15 years of education.^61^ Thus, on-the-job training has been the dominant approach for improving the quality of village doctors.^63^ In 2011, the average age of village doctors was 49 years,^52^ raising concerns that this ageing workforce would be less receptive to training, particularly training involving health information technology. Retraining of village doctors or their replacement with medical graduates emerges as a pivotal debate about the future of village doctors.

## Key messages—village doctors in China

Village doctors provide a rare opportunity of a huge cadre of health workers who are close to communities, and who also aspire to receive training and accreditation to improve the quality of their services.

However, there have been fluctuations in the policy environment determining their professional identity. Previously part-time farmers, part-time health workers, they are now regarded as full-time health workers. They have also seen transitions from public sector employment to private sector and back to a mixed model.

Village doctors' social and professional status is precarious, as they seek recognition and higher payment as public employees even as the government classifies them as rural farmers. Mandatory accreditation policies being considered could delegitimise village doctors, and remove communities' access to many village doctors who remain unlicensed.

### Integrating TCAM providers in the public health system in India

The government of India extends official recognition and support to certain Indian systems of medicine and homoeopathy. In the year 1995, the Department of Indian Systems of Medicine and Homoeopathy was established within the Ministry of Health and Family Welfare. In 2003, this department was renamed the Department of AYUSH, and governed the following types of TCAM: ayurveda, yoga and naturopathy, unani, siddha, homoeopathy and sowa rigpa (collectively called AYUSH). The National Rural Health Mission, launched in 2005, took an initiative to bring AYUSH into the public health mainstream in India, through recruitment of AYUSH personnel, and establishment of AYUSH centres of practice and field development. The Department of AYUSH was elevated to the Ministry of AYUSH in 2014. The ministry administers institutions and personnel of AYUSH education, training and practice.^64^ TCAM systems other than AYUSH are not included in the government's recognition.

One key question facing the Indian government is that whereas some types of TCAM systems are recognised and included in the public health system, others are not. Non-AYUSH TCAM systems, such as local health traditions in North-East India, and traditional medical systems not native to India, such as acupuncture, are not recognised or supported by the government, although codified, taught and practised by various private entities in India, and elsewhere. Consequently, government TCAM programmes promote the recognised AYUSH systems at the expense of more local systems, even in regions where they may be relatively alien.^65^

Second, while AYUSH has achieved official recognition, policy and planning to meet the workforce's needs are often contested. In policymaking by the government of India for a pluralistic health system, allopathic practice is disproportionately favoured in the funding allocations, administrative structures and employment conditions for healthcare practitioners. Although the number of AYUSH graduates is approximately equal to the number of biomedicine graduates from government-recognised educational institutions in India, the funding allocation for AYUSH was consistently as low as 1.3 to 2.7% of the entire budget for health for the majority of the past decade;^66^ the rest of the budget for health went to allopathic facilities, including institutions, infrastructure, supplies, research and personnel, revealing a glaring inequity. The elevation of the Department of AYUSH to a ministry in 2014 has set into motion some enhancements in budgetary allocation, and initiatives for the development of the fields of AYUSH.

In a seemingly ‘integrated’ national health system, power differentials between TCAM and allopathic providers impede the day-to-day functioning of mixed-system health facilities, and have adverse implications for the welfare and quality of work of the practitioners of different systems of medicine. Certain AYUSH providers in primary healthcare report pressure to prescribe allopathic medications, and manage allopathic outpatient consulting departments, from administrative supervisors who are usually allopathic doctors.^67^ ^68^ In a study of TCAM integration in the healthcare system in three Indian states, AYUSH providers reported widespread neglect of their concerns by administrators in favour of the concerns of their allopathic colleagues.^68^ ^69^

Further, AYUSH is broadly treated as a homogenous entity, although in reality it is a collection of diverse systems of medicine. The component systems of AYUSH have distinct requirements of infrastructural, technical, logistical and personnel support from the government for their establishment and operation in the public health structure of the country. However, planning, allocations and administrative structures and procedures do not account for these diverse needs. AYUSH has been incorporated into government health facilities in a largely homogenous manner all over the country, with imbalances in resources and support for some systems within the AYUSH ambit.

## Key messages—TCAM providers in India

The diverse TCAM sector in India includes a spectrum of providers from informal local healers to highly trained and government-accredited physicians in the AYUSH systems of medicine.

With at least as many healthcare providers and trained physicians in alternative systems of medicine, India's TCAM sector represents a massive, but only partially tapped, human resource for health.

The government of India's large-scale programmes to integrate TCAM providers in mainstream service delivery face many challenges. These include homogenisation of diverse systems including promoting mainstream TCAM systems among communities where they are not culturally accepted, and implicit hierarchies between different systems leading to ineffective deployment of personnel.

### Constrained employment opportunities for pharmacists in the Cambodian private sector

As in many low-income countries, the majority of the Cambodian population seeks healthcare in the private sector^19^ particularly for outpatient consulting, whereas 60% of all hospitalisations are in the public health sector.^70^ The major outflow of patients from the public to the private sector is to purchase medicines.^71^ ^72^ The private pharmacy sector includes sophisticated private hospitals, private clinics and licensed pharmacies, trained health workers as well as leased drug depots and retail shops manned by persons with no training in pharmacy.

The pharmacy sector is putatively regulated on the basis of owners' credentials and also on spatial distribution in keeping with population needs.^73^ While in 2008, only half the pharmacies and depot outlets in Cambodia were licensed, this proportion was declared to have risen to 100% in 2011.^20^ Nevertheless, numerous surveys and popular media reports over the years illuminate the continuing prevalence of unlicensed and illegal drug outlets.^73^ ^74^

Anthropologists have observed that local drug shops fulfil an important expectation from their customers, that is, the mixing of medicines, demonstrating expertise in healing, akin to the role played by traditional herbalists and the healers known as *kru khmer,* and distinct from formal allopathic practice.^75^ The fact that drug shops in rural areas allow financial flexibility, such as delayed payment or payment in kind, also seems important, and may approximate the relationship Khmer patients traditionally had with their *kru khmer.*^75^ On the other hand, physicians, whether they work in the public or private sector, own many of the drug outlets throughout the country, many without the pharmacy licence required for this by the government.^75^

Pharmacists in Cambodia hence experience formidable challenges to the consolidation of their professional and market niche. They are occupied in a twofold competition: against unlicensed drug shopkeepers who exploit regulatory loopholes to provide more flexible and consumer-friendly services, and against physician drug outlet owners, who have the advantage of higher social and professional status and popular acceptance. Pharmacists, at the conclusion of stringent higher education, are faced with very few jobs in the public sector, and competition in the private sector. While they often find better professional opportunities as sales representatives in pharmaceutical companies, they are not enabled to serve the population effectively, and to the best of their ability, in those positions.

## Key messages—pharmacists in Cambodia

Cambodia has a mixed public–private health system, with many patients using the private retail sector to purchase medicines.

Given the importance of safety and quality in the pharmacy sector, trained pharmacists in Cambodia have a potentially important role in healthcare delivery, yet there is a dearth of suitable jobs for them in the public sector.

Pharmacists also struggle to establish their market niche in the private sector, competing against doctors who own pharmacies on the one hand, and poorly regulated drug shopkeepers on the other. Formal training does not equip pharmacists to cater to patients' cultural expectations, and professional hierarchies help doctors dominate the pharmacy market.

## Conclusion: governing a mixed workforce

The diversity of health workforces in Asia, illustrated above, brings up a variety of complex governance questions. The case of the village doctors in China illustrates their contested identity across cadres, the public and private sectors and the formal and informal sectors. The case of TCAM practitioners and public sector services in India presents a stark picture of the power dynamics between different knowledge systems, and highlights how knowledge systems overlap in practice, and how recognition for some TCAM systems comes at the expense of others. The Cambodian pharmacists' struggles show how a mixed health system^76^ with a poorly resourced public sector and unregulated private sector does not automatically privilege training and formal recognition in its workforce. Underpinning each of these cases is the context of citizens' preferences and choices of health workers, not always consistent with mainstream policies.

The heterogeneity of the workforce offers a wide range of options for appropriate, safe and acceptable healthcare for the diverse populations of Asian LMICs; for the exercise of informed choice by users of health services; for diverse health providers to contribute in a complementary fashion to population health; and also the prospect of conservation of traditions and bodies of knowledge. However, undifferentiated policies for regulation, recognition and support cannot be expected to optimise the strengths of the heterogeneous health workforce. Differentiated approaches sensitive to the needs, competencies and regulatory requirements of different sections of the workforce are required to enhance population access to quality, safe and appropriate healthcare.

Policy for mixed human resources for health is a challenging arena, frequently entailing balancing acts between divergent policy priorities: ensuring access to comprehensive and locally acceptable health services; regulating healthcare quality and safety; and ensuring health workforce welfare and dignity. Policies may move entire sections of the workforce to new positions along various dimensions through legislative action, for example, by providing drug sellers or traditional birth attendants with tools and training in biomedicine, or by enlarging the scope of the practices of community health workers.

Ultimately, the formulation and implementation of policy needs to take into account the pluralistic nature of health systems, and acknowledge the *de facto* division of labour among the different segments. Appropriate policy responses also require attention to the epistemic divergences that may run counter to the assimilation of different bodies of knowledge and practice, and the unique administrative needs of different systems of knowledge and cadres of healthcare personnel.

## References

[1] World Health Organization. Framework on integrated people-centred health services. Sixty-ninth World Health Assembly A69/39, Provisional agenda item 16.1. 15 April 2016. http://apps.who.int/gb/ebwha/pdf_files/WHA69/A69_39-en.pdf?ua=1.

[2] Sheikh K, Ranson MK, Gilson L Explorations on people centredness in health systems. Health Policy Plan 2014;29(Suppl 2):ii1–5. 10.1093/heapol/czu082 25274634 PMC4202918

[3] Campbell J, Buchan J, Cometto G Human resources for health and universal health coverage: fostering equity and effective coverage. Bull World Health Organ 2013;91:853–63. 10.2471/BLT.13.118729 24347710 PMC3853950

[4] WHO. Health Workforce 2030: towards a global strategy on human resources for health 2015 http://www.who.int/hrh/documents/15-295Strategy_Report-04_24_2015.pdf.

[5] WHO. Health workforce 2016 http://www.who.int/hrh/tools/en/.

[6] Ramesh M, Wu X Realigning public and private health care in Southeast Asia. Pacific Rev 2008;21:171–87. 10.1080/09512740801990238

[7] Rafei UM, Sein UT Role of private hospitals in health care. Regional Health Forum WHO Southeast Asia Region 2006;5:1.

[8] Bose A Private health sector in India: is private health care at the cost of public health care?. BMJ 2005;331(7528):1338–9. 10.1136/bmj.331.7528.1338-c PMC1298900 16322030

[9] Sheikh K, George A India's health providers—diverse frontiers, disparate fortunes. In: Sheikh K, George A eds. Health providers in India: on the frontlines of change, New Delhi: Routledge, 2010, pp 1–14.

[10] Pedersen D, Baruffati V Healers, deities, saints and doctors: elements for the analysis of medical systems. Soc Sci Med 1989;29:487–96. 10.1016/0277-9536(89)90194-9 2756435

[11] Sudhinaraset M, Ingram M, Lofthouse HK What is the role of informal healthcare providers in developing countries? A systematic review. PLoS ONE 2013;8:e54978 10.1371/journal.pone.0054978 23405101 PMC3566158

[12] Shah NM, Brieger WR, Peters DH Can interventions improve health services from informal private providers in low and middle-income countries?: a comprehensive review of the literature. Health Policy Plan 2011;26:275–87. 10.1093/heapol/czq074 21097784

[13] BHW (Bangladesh Health Watch). Health workforce in Bangladesh: Who constitutes the health care system? The state of health in Bangladesh 2007. Dhaka: James P Grant School of Public Health, BRAC University, 2007.

[14] Kanjilal B, Mondol S, Samanta T A parallel health care market: rural medical practitioners in West Bengal, India. Research brief. Jaipur: Institute of Health Management Research, 2007.

[15] Liu Y, Berman P, Yip W Health care in China: the role of non-government providers. Health Policy 2006;77:212–20. 10.1016/j.healthpol.2005.07.002 16112771

[16] Hong Ha NT, Berman P, Larsen U Household utilization and expenditure on private and public health services in Vietnam. Health Policy Plan 2002;17:61–70. 10.1093/heapol/17.1.61 11861587

[17] Ozawa S, Walker DG Comparison of trust in public vs private health care providers in rural Cambodia. Health Policy Plan 2011;26:120–9. 10.1093/heapol/czr045 21729914

[18] Wang H, McEuen M, Mize L Private sector health in Indonesia: a desk review. Bethesda, MD: Health Systems 20/20 project, Abt Associates Inc, 2009.

[19] National Institute of Statistics, Directorate General for Health, and ICF Macro, 2011. Cambodia Demographic and Health Survey 2010 Phnom Penh, Cambodia and Calverton, Maryland, USA: National Institute of Statistics, Directorate General for Health, and ICF Macro.

[20] WHO and Ministry of Health Cambodia. Health Service Delivery Profile Cambodia 2012 2012 http://www.wpro.who.int/health_services/service_delivery_profile_cambodia.pdf.

[21] Meessen B, Bigdeli M, Chheng K Composition of pluralistic health systems. How much can we learn from household surveys? An exploration in Cambodia. Health Policy Plan 2011;26:i30–44. 10.1093/heapol/czr026 21729915

[22] Basu S, Andrews J, Kishore S Comparative performance of private and public healthcare systems in low- and middle-income countries: a systematic review. PLoS Med 2012;9:e1001244 10.1371/journal.pmed.1001244 22723748 PMC3378609

[23] Sheikh K, Saligram PS, Hort K. What explains regulatory failure? Analysing the architecture of health care regulation in two Indian states. Health Policy Plan 2015;30:39–55. 10.1093/heapol/czt095 24342742

[24] Ahmed SM, Hossain MA, Chowdhury MR Informal sector providers in Bangladesh: how equipped are they to provide rational health care? Health Policy Plan 2009;24:467–78. 10.1093/heapol/czp037 19720721

[25] Chalker JC Interventions for improved prescribing and dispensing in Nepal, Thailand and Vietnam. Thesis (PhD) Stockholm: Karolinska Institutet, 2003.

[26] Chuc N Towards good pharmacy practice in Hanoi-a multi-intervention study in private sector. Thesis (PhD) Stockholm: Karolinska Institutet, 2002.

[27] Bloom G, Lucas H Health and Poverty in sub-Saharan Africa. IDS Working Paper no. 103 Brighton, UK: Institute of Development Studies, 2000.

[28] Leslie C Medical pluralism in world perspective. Soc Sci Med Med Anthropol 1980;14B:191–5. 7209590 10.1016/0160-7987(80)90044-7

[29] George A, Iyer A Unfree markets: socially embedded informal health providers in northern Karnataka, India. Soc Sci Med 2013;96:297–304. 10.1016/j.socscimed.2013.01.022 23484865

[30] Standing H, Chowdhury AM Producing effective knowledge agents in a pluralistic environment: what future for community health workers? Soc Sci Med 2008;66:2096–107. 10.1016/j.socscimed.2008.01.046 18342421

[31] MAQARI Team. Mapping medical providers in rural India: four key trends [Internet] Centre for Policy Research. New Delhi http://cprindia.org/sites/default/files/policy%20brief_1.pdf.

[32] De Costa A, Diwan V ‘Where is the public health sector?’ Public and private sector healthcare provision in Madhya Pradesh, India. Health Policy 2007;84:269–76. 10.1016/j.healthpol.2007.04.004 17540472

[33] Iqbal M, Wahed T, Hanifi SMA Lessons from an intervention programme to make informal healthcare providers effective in a rural area of Bangladesh. In: Bloom G, Kanjilal B, Henry L, eds. Transforming Health Markets in Asia and Africa Improving quality and access for the poor. Routledge, Oxford, 2013, pp 35–57.

[34] Leslie C ed. Asian medical systems: a comparative study. Berkeley, Los Angeles, London: University of California Press, 1976.

[35] Sydara K, Gneunphonsavath S, Wahlström R Use of traditional medicine in Lao PDR. Compliment Ther Med 2005;13:199–205. 10.1016/j.ctim.2005.05.004 16150374

[36] Gogtay NJ, Bhatt HA, Dalvi SS The use and safety of non-allopathic Indian Medicines. Drug Saf 2002;25:1005–19. 10.2165/00002018-200225140-00003 12408732

[37] Ladinsky JL, Volk ND, Robinson M The influence of traditional medicine in shaping medical care practices in Vietnam today. Soc Sci Med 1987;25:1105–10. 10.1016/0277-9536(87)90351-0 3317871

[38] Ghani A Present state-of-the art of traditional medicine practice in Bangladesh. In: Mosaddegh M, Naghibi F eds. Traditional medicine and material medica Vol 1. Tehran: Traditional Medicine and Materia Medica Research Centre, Shahid Behest University of Medical Sciences, 2002:59–70. http://www.itmrc.org/publication/ch_5.htm (accessed 19 Jan 2014).

[39] Lakshmi JK, Nambiar D, Narayan V Cultural consonance, constructions of science, and co-existence: a review of the integration of traditional, complementary, and alternative medicine in low- and middle-income countries. Health Policy Plan 2015;30:1067–77. 10.1093/heapol/czu096 25171821 PMC4559111

[40] Torri MC Perceptions of the use of complementary therapy and siddha medicine among rural patients with HIV/AIDS: a case study from India. Int J Health Plann Manage 2013;28:63–84. 10.1002/hpm.2133 22887471

[41] Bodeker G Lessons on integration from the developing world's experience. BMJ 2001;322:164–7. 10.1136/bmj.322.7279.164 11159579 PMC1119421

[42] Gawde SR, Shetty YC, Pawar DB Knowledge, attitude, and practices toward ayurvedic medicine use among allopathic resident doctors: a cross-sectional study at a tertiary care hospital in India. Perspect Clin Res 2013;4:175–80. 10.4103/2229-3485.115380 24010059 PMC3757582

[43] Singh J, Raje N The rise of western medicine in India. Lancet 1996;348:1598 10.1016/S0140-6736(05)66231-9 8950923

[44] Sharma DC India to promote integration of traditional and modern medicine. Lancet 2001;358:1524 10.1016/S0140-6736(01)06630-2 11705580

[45] Chung VC, Hillier S, Lau CH Referral to and attitude towards traditional Chinese medicine amongst western medical doctors in postcolonial Hong Kong. Soc Sci Med 2011;72:247–55. 10.1016/j.socscimed.2010.10.021 21145150

[46] Lakshmi JK Delhi Medical Council ruling on cross-system practice by practitioners of AYUSH. Natl Med J India 2016;29:114. 27586227

[47] Fulton BD, Scheffler RM, Sparkes SP Health workforce skill mix and task shifting in low income countries: a review of recent evidence. Hum Resour Health 2011;9:1 10.1186/1478-4491-9-1http://www.human-resources-health.com/content/9/1/1 (accessed 19 Jan 2014). 21223546 PMC3027093

[48] Arifeen SE, Christou A, Reichenbach L Community-based approaches and partnerships: innovations in health-service delivery in Bangladesh. Lancet 2013;382:2012–26 10.1016/S0140-6736(13)62149-2. 24268607

[49] Budlender D Time use studies and unpaid care work. New York: Routledge, 2010.

[50] Nambiar D, Sheikh K How a technical agency helped scale up a community health worker program: an exploratory study in Chhattisgarh State, India. Health Systems & Reform 2016;2:123–34. 10.1080/23288604.2016.1148802 31514641

[51] Hesketh T, Zhu WX Health in China: the healthcare market. BMJ 1997;314:1616 10.1136/bmj.314.7094.1616 9186178 PMC2126808

[52] Xu H, Zhang W, Zhang X Longitudinal study of rural health workforce in five countries in China: research design and baseline description. Hum Resour Health 2103;11:17 10.1186/1478-4491-11-17 PMC3656804 23642224

[53] Zhang D, Unschuld PU China's barefoot doctor: past, present, and future. Lancet 2008;372:1865–7. 10.1016/S0140-6736(08)61355-0 18930539

[54] Huang Q, Jing S, Huang D Basic situation of village doctors in China during “the eighth five year project”. Practical Rural Doctors J (in Chinese) 1995;6:4–9.

[55] Guo Q, Wang Z, Yan H A survey and analysis on village doctors in 46 poor counties in Western China. China Public Health(in Chinese) 2003;22:51–2.

[56] Xie Y, Zhao J, Zhu L A survey of village doctors and their health service status. Chinese Primary Health Care (in Chinese) 2003;17.

[57] Hipgrave D, Guo S, Mu Y Chinese-style decentralization and health system reform. PLoS Med 2012;9:e1001337 10.1371/journal.pmed.1001337 23139644 PMC3491007

[58] Liu P, Zhao J The difficulty and solution of village doctors in the new health reform. Health Econ Res (in Chinese) 2013;9:23–4.

[59] Zhang S, Zhang W, Zhou H How China's new health reform influences village doctors’ income structure: evidence from a qualitative study in six counties in China. Hum Resour Health 2015;13:26 10.1186/s12960-015-0019-1. 25940189 PMC4440293

[60] State Council. The Guidance of the State Council to Launch the New Rural Social Pension Insurance System (in Chinese). The State Council of China (2009), Policy Document #75, Beijing, China 2009.

[61] Ministry of Health. 2012 China Health Statistics Yearbook (in Chinese). Beijing: Beijing Union Medical University Press, 2012.

[62] Anand S, Fan VY, Zhang J Health system reform in China 5: China's human resources for health: quantity, quality, and distribution. Lancet 2008;372:1774–81. 10.1016/S0140-6736(08)61363-X 18930528

[63] State Council. The Guideline for Improving the Village Doctors by State Council (in Chinese). The State Council of China (2011), Policy Document #31, Beijing, China 2011.

[64] Ministry of AYUSH. Welcome to AYUSH 2015 http://indianmedicine.nic.in/

[65] Albert S, Porter J Is ‘mainstreaming AYUSH’ the right policy for Meghalaya, northeast India? BMC Complement Altern Med 2015;15:288 10.1186/s12906-015-0818-x 26283420 PMC4539927

[66] Priya R AYUSH and public health: democratic pluralism and the quality of health services. In: Sujatha V, Abraham L, Medical pluralism in contemporary India. eds. Orient Blackswan, 2012, pp 103–29.

[67] Lakshmi JK Less equal than others? Experiences of AYUSH medical officers in primary health centres in Andhra Pradesh. Indian J Med Ethics 2012;9:18–21. 22319847 10.20529/IJME.2012.005

[68] Josyula KL, Sheikh K, Nambiar D *“Getting the water-carrier to light the lamps”*: discrepant role perceptions of traditional, complementary, and alternative medical practitioners in government health facilities in India. Soc Sci Med 2016;166:214–22. 10.1016/j.socscimed.2016.08.038 27575933 PMC5034848

[69] Nambiar D, Narayan VV, Josyula LK Experiences and meanings of integration of TCAM (Traditional, Complementary and Alternative Medical) providers in three Indian states: results from a cross-sectional, qualitative implementation research study. BMJ Open 2014;4:e005203 10.1136/bmjopen-2014-005203 PMC4248091 25424993

[70] World Bank Group. Cambodia's rural health markets and the quality of care. Knowledge brief: health, nutrition and population global practice: 93386 Washington, DC: World Bank Group, 2014 http://documents.worldbank.org/curated/en/514181468213569828/Cambodias-rural-health-markets-and-the-quality-of-care

[71] Yanagisazwa S, Mey V, Wakai S Comparison of health seeking behaviour between poor and better-off people after health sector reform in Cambodia. Public Health 2004;118:21–30. 10.1016/S0033-3506(03)00140-9 14643624

[72] Chhea C, Warren N, Manderson L Health worker effectiveness and retention in rural Cambodia. Rural Remote Health 2010;10:1391. 20701412

[73] Sievleang LY A study on restricted regulation in pharmacy market in Cambodia. KDI School of Public Policy and Management, 2014 http://www.slideshare.net/Sievleang/sievleang-pharmacy-pptaccessing.

[74] Meyn C. Drugs sector outpacing regulation. The Phnom Penh post. Mon, 23 November 2009 http://www.phnompenhpost.com/business/drugs-sector-outpacing-regulation

[75] Trankell IB, Ovesen J Pharmacists and other drug providers in Cambodia: identities and experiences. In: Maynard A eds. “Medical identities: health, wellbeing and personhood”. Berghahn Books, 2007, pp 36–60.

[76] Nishtar S The mixed health systems syndrome. Bull World Health Organ 2010;88(1):74–5. 10.2471/BLT.09.067868 20428356 PMC2802440

